# Pharmacological Modulation of Mitochondrial Ca^2+^ Content Regulates Sarcoplasmic Reticulum Ca^2+^ Release via Oxidation of the Ryanodine Receptor by Mitochondria-Derived Reactive Oxygen Species

**DOI:** 10.3389/fphys.2018.01831

**Published:** 2018-12-21

**Authors:** Shanna Hamilton, Radmila Terentyeva, Tae Yun Kim, Peter Bronk, Richard T. Clements, Jin O-Uchi, György Csordás, Bum-Rak Choi, Dmitry Terentyev

**Affiliations:** ^1^Department of Medicine, The Warren Alpert Medical School of Brown University, Rhode Island Hospital, Cardiovascular Research Center, Providence, RI, United States; ^2^Department of Surgery, The Warren Alpert Medical School of Brown University, Rhode Island Hospital, Cardiovascular Research Center, Providence, RI, United States; ^3^Vascular Research Laboratory, Providence Veterans Affairs Medical Center, Providence, RI, United States; ^4^Lillehei Heart Institute University of Minnesota, Cancer and Cardiovascular Research Building, Minneapolis, MN, United States; ^5^Department of Pathology, Anatomy and Cell Biology, Thomas Jefferson University, Philadelphia, PA, United States

**Keywords:** mitochondria, reactive oxygen species, ryanodine receptor, hypertrophy, ventricular arrhythmia, Ca^2+^-induced Ca^2+^ release

## Abstract

In a physiological setting, mitochondria increase oxidative phosphorylation during periods of stress to meet increased metabolic demand. This in part is mediated via enhanced mitochondrial Ca^2+^ uptake, an important regulator of cellular ATP homeostasis. In a pathophysiological setting pharmacological modulation of mitochondrial Ca^2+^ uptake or retention has been suggested as a therapeutic strategy to improve metabolic homeostasis or attenuate Ca^2+^-dependent arrhythmias in cardiac disease states. To explore the consequences of mitochondrial Ca^2+^ accumulation, we tested the effects of kaempferol, an activator of mitochondrial Ca^2+^ uniporter (MCU), CGP-37157, an inhibitor of mitochondrial Na^+^/Ca^2+^ exchanger, and MCU inhibitor Ru360 in rat ventricular myocytes (VMs) from control rats and rats with hypertrophy induced by thoracic aortic banding (TAB). In periodically paced VMs under β-adrenergic stimulation, treatment with kaempferol (10 μmol/L) or CGP-37157 (1 μmol/L) enhanced mitochondrial Ca^2+^ accumulation monitored by mitochondrial-targeted Ca^2+^ biosensor mtRCamp1h. Experiments with mitochondrial membrane potential-sensitive dye TMRM revealed this was accompanied by depolarization of the mitochondrial matrix. Using redox-sensitive OMM-HyPer and ERroGFP_iE biosensors, we found treatment with kaempferol or CGP-37157 increased the levels of reactive oxygen species (ROS) in mitochondria and the sarcoplasmic reticulum (SR), respectively. Confocal Ca^2+^ imaging showed that accelerated Ca^2+^ accumulation reduced Ca^2+^ transient amplitude and promoted generation of spontaneous Ca^2+^ waves in VMs paced under ISO, suggestive of abnormally high activity of the SR Ca^2+^ release channel ryanodine receptor (RyR). Western blot analyses showed increased RyR oxidation after treatment with kaempferol or CGP-37157 vs. controls. Furthermore, in freshly isolated TAB VMs, confocal Ca^2+^ imaging demonstrated that enhancement of mitochondrial Ca^2+^ accumulation further perturbed global Ca^2+^ handling, increasing the number of cells exhibiting spontaneous Ca^2+^ waves, shortening RyR refractoriness and decreasing SR Ca^2+^ content. In *ex vivo* optically mapped TAB hearts, kaempferol exacerbated proarrhythmic phenotype. On the contrary, incubation of cells with MCU inhibitor Ru360 (2 μmol/L, 30 min) normalized RyR oxidation state, improved intracellular Ca^2+^ homeostasis and reduced triggered activity in *ex vivo* TAB hearts. These findings suggest facilitation of mitochondrial Ca^2+^ uptake in cardiac disease can exacerbate proarrhythmic disturbances in Ca^2+^ homeostasis via ROS and enhanced activity of oxidized RyRs, while strategies to reduce mitochondrial Ca^2+^ accumulation can be protective.

## Introduction

Sudden cardiac death remains the leading global cause of mortality, and over half of patients with heart failure (HF) die suddenly due to the development of ventricular arrhythmia (Benjamin et al., 2018). Arrhythmogenesis in the failing heart is often linked to enhanced Ca^2+^-dependent triggered activity, in the form of early and delayed afterdepolarizations (Landstrom et al., 2017). These abnormal electrical activities arise in part as a consequence of untimely and dysregulated Ca^2+^ release from the sarcoplasmic reticulum (SR), through SR Ca^2+^ release channel, the ryanodine receptor (RyR). Abnormal activity of RyR leads to increased Ca^2+^ leak and promotes the generation of spontaneous Ca^2+^ waves (SCWs), that can subsequently propagate to trigger organ-wide arrhythmia (Bers, 2002).

Mitochondria play an essential role in cardiac Ca^2+^ homeostasis in physiological conditions (Kwong et al., 2015; Luongo et al., 2017). Excitation-contraction coupling consumes large amounts of ATP and mitochondria increase oxidative phosphorylation to meet increased metabolic demand. Influx of Ca^2+^ into the mitochondria is critical for the availability of ATP as major enzymes in the tricarboxylic acid cycle are activated by Ca^2+^. Mitochondria are in close spatial proximity to the SR (Dorn and Scorrano, 2010; Eisner et al., 2013; Lu et al., 2013; Seidlmayer et al., 2016; Lopez-Crisosto et al., 2017; Csordás et al., 2018), and it is well established that during higher workload, there is an elevation of cystolic Ca^2+^ concentration in ventricular myocytes (VMs) that transpires to a small and slow rise in mitochondrial Ca^2+^ concentration ([Ca^2+^]_m_), leading to enhanced energy production (Brandes and Bers, 1997; Luongo et al., 2015). Influx of Ca^2+^ through the mitochondrial Ca^2+^ uniporter (MCU) channel complex is driven largely by the negative membrane potential across the inner mitochondrial membrane (Kirichok et al., 2004; Baughman et al., 2011; De Stefani et al., 2011). Conversely, mitochondrial efflux mainly occurs via the mitochondrial Na/Ca^2+^/Li^+^-exchanger (NCLX) (Palty et al., 2010; Boyman et al., 2013; Luongo et al., 2017).

Mitochondria are a major source of reactive oxygen species (ROS) in the myocyte, and while an increase in oxidative stress is a prerequisite for many cellular stress responses, excessive ROS production in cardiac disease contributes to ventricular arrhythmogenesis by altering the function of multiple ion channels and transporters (Zima and Blatter, 2006; Niggli et al., 2013; Wagner et al., 2013). RyRs are highly sensitive to ROS, and contain multiple redox-sensitive cysteine residues (Zima and Blatter, 2006). Cysteine thiol oxidation of RyR increases channel activity, and many groups including ours have previously established that increased RyR oxidation in VMs from diseased hearts promotes proarrhythmic spontaneous SR Ca^2+^ release in the form of propagating Ca^2+^ waves that underlie increased triggered activity (Terentyev et al., 2008; Belevych et al., 2009; Cooper et al., 2013; Kyrychenko et al., 2013; Bovo et al., 2018). Scavenging of mitochondrial ROS was shown to improve Ca^2+^ homeostasis and attenuate arrhythmic potential in multiple models of cardiac disease including HF, hypertrophy, diabetic cardiomyopathy or aging (Mochizuki et al., 2007; Terentyev et al., 2008; Belevych et al., 2012; Cooper et al., 2013; Luo et al., 2013; Joseph et al., 2016; Kim et al., 2017).

Given the contribution of mitochondrial dysfunction to multiple cardiac disease states, maintaining mitochondrial Ca^2+^ homeostasis remains an attractive therapeutic target (Dietl and Maack, 2017). In conditions with defective intracellular Ca^2+^ and Na^2+^ homeostasis such as in models of HF, increasing [Ca^2+^]_m_ above a specific threshold was suggested to improve metabolism and substrate utilization, as well as reduce oxidative stress and ROS overload in the myocyte (Liu and O’Rourke, 2008; Kolhaas et al., 2010; Liu et al., 2014). More recently, Schweitzer et al. (2017) suggested that pharmacological enhancement of [Ca^2+^]_m_ suppressed arrhythmia in a model of catecholaminergic polymorphic ventricular tachycardia (CPVT), a condition characterized by mutations in the RyR macromolecular complex that renders channels hyperactive. Given mitochondria are in close proximity to SR Ca^2+^ release sites, it has been proposed that increasing mitochondrial Ca^2+^ uptake may improve buffering capacity (Seguchi et al., 2005; Drago et al., 2012; Zhao et al., 2013), thereby limiting local Ca^2+^ release events, Ca^2+^ sparks, which would result in a decrease in generation and propagation velocity of proarrhythmic SCWs. Conversely, a reduction of mitochondrial Ca^2+^ uptake may serve as an anti-arrhythmic strategy. In models of HF and ischemia-reperfusion, pathological mitochondrial dysfunction and mitochondria Ca^2+^ overload contribute to oxidative stress and cell death (Santulli et al., 2015). Pharmacological inhibition (García-Rivas Gde et al., 2006; Xie et al., 2018) or genetic ablation (Kwong et al., 2015; Luongo et al., 2015) of MCU, as well as conditional NCLX overexpression (Luongo et al., 2017) has been shown to protect against ischemia-induced myocyte injury, the development Ca^2+^-dependent arrhythmia and the progression of HF.

In the present study, we aimed to determine the effects of pharmacological facilitation of [Ca^2+^]_m_ accumulation and inhibition of mitochondrial Ca^2+^ uptake on intracellular Ca^2+^ homeostasis and arrhythmic potential using rat model of cardiac hypertrophy induced by thoracic aortic banding (TAB). To achieve this goal, we utilized whole heart optical mapping, genetically encoded ROS and mitochondrial Ca^2+^ biosensors, confocal microscopy and biochemistry to dissect the influence of MCU enhancer kaempferol and NCLX inhibitor CGP-37157 on intracellular Ca^2+^ cycling, in both healthy and hypertrophic VMs. Our results suggest that enhancement of mitochondrial Ca^2+^ accumulation in either setting elevates mitochondrial ROS emission, increasing oxidation of RyR and aberrant spontaneous Ca^2+^ release. Attenuating mitochondrial Ca^2+^ uptake serves as an anti-arrhythmic treatment in hypertrophic hearts, whereby triggered activity was reduced by pharmacological inhibition of MCU with Ru360.

## Materials and Methods

### Ethics Statement

Procedures involving animals were approved by The Rhode Island Hospital Institutional Animal Care and Use Committee and followed the Guide for the Care and Use of Laboratory Animals published by the US National Institutes of Health (NIH Publication No. 85-23, revised 2011).

### Generation of Adenoviral Constructs

The mitochondrial targeting sequence cytochrome C oxidase subunit IV was fused as the N-terminal of the coding sequence of plasmid RCamp1h (Akerboom et al., 2013) to create a probe to monitor intra-mitochondrial Ca^2+^. pC1-HyPer-3 was a gift from Vsevolod Belousov (Addgene plasmid # 42131). The mitochondrial localization sequence of mAKAP1 followed by a linker was fused as the N-terminus of the coding sequence of pC1-HyPer-3 (Burns-Hamuro et al., 2003; DiPilato et al., 2004; Bilan et al., 2013). This enables anchoring of the probe to the outer mitochondrial membrane (OMM) to measure H_2_O_2_ at the mitochondrial surface, and the subsequent viral construct is thus referred to as OMM-HyPer. ERroGFP_iE_pCDNA3 was a gift from David Ron (Addgene plasmid # 47954). The ERroGFP_iE probe is targeted to the endoplasmic reticulum (ER) by the cleavable signal peptide and C-terminal KDEL ER retrieval signal (Avezov et al., 2013). Adenovirus carrying plasmid constructs were generated utilizing the ViraPower Gateway expression system (Thermo Fisher Scientific, Waltham, MA, United States). Briefly, coding regions were cloned into the pENTR^TM^ 1A entry vector, and recombined into pAd/CMV/V5-DEST^TM^ destination vector by LR recombinase reaction. Once sequence-verified, destination vector plasmids were digested with restriction enzyme *PacI* and transfected into HEK293A cells using Lipofectamine^TM^ 2000 (Thermo Fisher Scientific). Adenoviral stock titer was determined using the Adeno-X qPCR Titration Kit (Takara Bio USA, Inc., Mountain View, CA, United States).

### Myocyte Isolation and Primary Culture

Myocytes were isolated from male 9- to 12-week-old Sprague-Dawley rats (controls) from Harlan Laboratories (Indianapolis, IN, United States). Male Sprague-Dawley rats with TAB surgery were purchased from Charles River Laboratories (Wilmington, MA, United States). Animals were shipped 5–7 days after surgery and acclimatized for 3–4 weeks in the Rhode Island Hospital animal facility. Experiments were performed 4–5 weeks after aortic banding procedure.

Bilateral thoracotomy was performed on euthanized rats and the heart plunged into ice cold Tyrode’s solution. The hearts were mounted on a Langendorff apparatus and retrogradely perfused with Tyrode solution (Terentyev et al., 2009) containing collagenase II (Worthington Biochemical Corp., Lakewood, NJ, United States) at 37°C for 16–17 min. Ventricles were minced and placed in a 37°C water bath shaker in collagenase solution. Isolated VMs were plated onto laminin-coated glass coverslips in 24-well plates.

For experiments with cultured control and TAB rats VMs, myocytes were cultured in serum-free medium 199 (Thermo Fisher Scientific), supplemented with 25 mmol/L NaHCO_3_, 10 mmol/L HEPES, 5 mmol/L creatine, 5 mmol/L taurine, 10 μ/mL penicillin, 10 μg/mL streptomycin and 10 μg/mL gentamycin (pH 7.3). Unattached cells were removed after 1 h and remaining VMs were cultured for 48 h. Cultured VMs were infected with adenoviruses at multiplicity of infection (MOI) of 10 for all described constructs. Myocytes were cultured at 37°C in 95% air, 5% CO_2_ for 36–48 h before analysis.

### Pharmacological Modifiers of Mitochondrial Ca^2+^ Uptake, Mitochondrial ROS, and RyR Activity

Kaempferol directly activates MCU (Montero et al., 2004; Vay et al., 2007), and was obtained from Millipore Sigma (Burlington, MA, United States), used at 10 μmol/L. CGP-37571 inhibits NCLX (Liu and O’Rourke, 2008; Kolhaas et al., 2010; Liu et al., 2010), and was obtained from Millipore Sigma, used at 1 μmol/L. SB 202190 is an inhibitor of p38 mitogen-activated protein (MAP) kinase, has also been shown to activate MCU (Montero et al., 2004), and was obtained from Millipore Sigma, used at 30 μmol/L. Ru360 specifically inhibits mitochondrial Ca^2+^ uptake through MCU (Matlib et al., 1998; García-Rivas Gde et al., 2006), and was obtained from Millipore Sigma, used at 2 μmol/L. MitoTEMPO, a specific scavenger of mitochondrial superoxide was obtained from Millipore Sigma, used at 20 μmol/L. Dantrolene, an RyR antagonist (Kobayashi et al., 2009; Maxwell et al., 2012) was obtained from Millipore Sigma and used at 2 μmol/L.

### Confocal Imaging

Confocal imaging was performed using a Leica SP5 II confocal microscope equipped with 63 × 1.4 numerical aperture oil objective in linescan and x–y mode. All confocal imaging experiments were performed under β-adrenergic stimulation with 50 nmol/L isoproterenol (ISO, Millipore Sigma). Control VMs were paced via field stimulation at 2 Hz, while TAB VMs were paced at 0.5 Hz using extracellular platinum electrodes. Myocytes were studied in Tyrode’s solution (Terentyev et al., 2009). Confocal imaging data were analyzed using Leica Software, Origin 8.0 (OriginLab, Northampton, MA, United States) and ImageJ (National Institutes of Health, Bethesda, MA, United States).

Intact VMs were loaded with Rhod-2 AM (Thermo Fisher Scientific) at room temperature for 12 min, followed by a 10 min wash. Rhod-2 was excited using 543 nm line of HeNe laser and fluorescence emission was collected at 560–660 nm wavelengths in linescan mode at 200 Hz sampling rate. Calcium transients were recorded at room temperature. To test for the propensity of triggered activity, VMs were paced for 20 s and latency between the last pacing stimulus and the subsequent SCW was calculated. To assess SR Ca^2+^ load, 10 mmol/L caffeine was applied at the end of experiments. The data is presented as Δ*F*/*F*_0_, where *F*_0_ is basal fluorescence and Δ*F* = *F* – *F*_0_.

Biosensor mtRCamp1h was excited using 543 nm line of HeNe laser and fluorescence emission was collected at 560–660 nm wavelengths. For permeabilized VM experiments, myocytes were saponin-permeabilized (0.001%) and equilibrated with a solution containing thapsigargin (10 μmol/L), cytochalasin D (10 μmol/L), FCCP (20 μmol/L), and ionomycin (5 μmol/L). Tyrode’s solution containing Ca^2+^ buffer EGTA (2 mmol/L) was applied to obtain minimum mtRCamp1h fluorescence. Maximum fluorescence was achieved by application of Ca^2+^ (100 μmol/L). The data is presented as as Δ*F*/*F*_0_, where *F*_0_ is basal fluorescence and Δ*F* = *F* – *F*_0_. Biosensors ERroGFP_iE and OMM-HyPer were excited using 488 nm line of Argon laser and fluorescence emission was collected at 500–550 nm wavelengths, measured in x–y mode. Maximum fluorescence (*F*max) was obtained by application 200 μmol/L DTDP and minimum fluorescence was obtained by application ROS scavenger DTT (5 mmol/L). Data is presented as a percentage of Δ*F*/Δ*F*max where Δ*F* = *F* – *F*min, and Δ*F*max = *F*max–*F*min. Mitochondrial membrane potential was monitored with the voltage-sensitive fluorescent indicator, tetramethylrhodamine, methyl ester (TMRM; Thermo Fisher Scientific). Isolated VMs were loaded with 20 μmol/L for 1 min and washed thoroughly prior to imaging. TMRM was excited using 543 nm line of HeNe laser and fluorescence emission was collected at 560–660 nm wavelengths, measured in x–y mode. Fluorescence of TMRM was normalized to the minimum fluorescence signal obtained by application of mitochondrial uncoupler, carbonyl *p*-(trifluoromethoxy) phenylhydrazone (FCCP, 50 μmol/L). For experiments with mitoTEMPO, isolated VMs were pretreated with mitoTEMPO (20 μmol/L, 30 min), before loading with TMRM as described.

The emission of ROS was measured in isolated VMs in Tyrode solution using MitoSOX Red mitochondrial superoxide indicator (Thermo Fisher Scientific; 20 μmol/L, 30 min loading). The indicator was excited with 514 nm line of an argon laser and emission was collected at 560–660 nm, measured in x–y mode. Fluorescence of MitoSOX was normalized to the maximum fluorescence signal obtained by application of peroxide H_2_O_2_ (10 mmol/L).

### Oxidation of RyR and Western Blotting

Freshly isolated control or TAB rat VMs were treated with ISO (50 nmol/L, 5 min total), kaempferol (10 μmol/L, 5 min total) or CGP (1 μmol/L, 10 min total) and paced for 1 min at 2 Hz at room temperature before immediate lysis in lysis buffer from Cell Signaling (Danvers, MA, United States, Cat#9803S), supplemented with phosphatase (Calbiochem, San Diego, CA, United States, Cat#524625) and protease inhibitor cocktails (Millipore Sigma, Cat#P8340) as described previously (Terentyev et al., 2014). For co-immunoprecipitation, Pierce Co-immunoprecipitation Kit (Thermo Fisher Scientific, Cat#26149) was used. Lysate (500 μl) was pre-cleared with Control Agarose Resin for 30 min at 4°C, centrifuged at 1,000 × *g* for 1 min. Flow-through was incubated with antibody-coupled resin (anti-RyR2, Thermo Fisher Scientific, Cat#MA3-916 and negative control antibody, normal mouse IgG, Santa Cruz Biotechnology, Cat#sc-2025) for 2 h at 4°C. Columns were washed three times. Protein complexes were eluted with elution buffer provided in the kit. To determine oxidation of RyR, the Oxidized Protein Western Blot Kit (Abcam, Cambridge, MA, United States, Cat#ab178020) was used, whereby carbonyl groups of immunoprecipitated RyR2 were derivatized to 2,4 dinitrophenylhydrazone (DNP) by reaction with 2,4 dinitrophenylhydrazine. For control we used Derivatization Control Solution, provided in the kit. The DNP-RyR2 protein samples were separated on 4–20% Mini-PROTEAN TGX gels (Bio-Rad Laboratories, Hercules, CA, United States, Cat#456-1094) and DNP-associated signal assessed by the kit-provided anti-DNP primary antibody and anti-RyR2 (Thermo Fisher Scientific, Cat#MA3-916), followed by HRP-conjugated secondary antibody and anti-mouse lgG(H+L) HRP secondary antibody (Promega, Madison, WI, United States, Cat#W4021). Abcam antibodies (Cat#ab57602 and Cat#ab101055) were used to assess expression levels of mitofusin 1 and mitofusin 2. Anti-glyceraldehyde 3-phosphate dehydrogenase (GAPDH) antibodies were used for loading control (Abcam, Cat#ab8245). Blots were developed with ECL (Bio-Rad Laboratories) and quantified using ImageJ and Origin 8 software.

### *Ex vivo* Optical Mapping

Beating hearts were harvested from anesthetized TAB rats via thoracotomy and were retrogradely perfused through the aorta in a Langendorff perfusion system (Radnoti Glass Technology, Monrovia, CA, United States) with (in mmol/L): 130 NaCl, 24 NaHCO_3_, 1.0 MgCl_2_, 5.0 KCl, 1.2 NaH_2_PO_4_, 5 dextrose, and 1 CaCl_2_, at pH 7.4, gassed with 95% O_2_ and 5% CO_2_. Constant flow perfusion was set to 10 mL/min with a peristaltic pump. Hearts were placed in a water-heated chamber to maintain temperature at 37 ± 0.2°C, and 5 μmol/L blebbistatin was added to perfusate to reduce movement artifact. Hearts were loaded with Ca^2+^ indicator Rhod-2 AM (Thermo Fisher Scientific), using 25 μL of stock solution (1 mg/mL of DMSO) delivered through a bubble trap, above the aortic cannula. The ECGs were continuously monitored with a Powerlab system (AD Instrument, Colorado Springs, CO, United States). The optical apparatus has been described previously (Kim et al., 2015). Fluorescence images of Rhod-2 signal were recorded from the anterior surface of the heart using a CMOS camera (100 × 100 pixels, 2000 frames/sec, 1.5 cm × 1.5 cm field of view, Ultima-L, SciMedia, Japan). Drugs (kaempferol and Ru360) were perfused for 20–30 min and ISO (50 nmol/L) was added to investigate the effect of drugs on VT/VF induction in TAB hearts. The fluorescence (F) from Rhod-2 was normalized with ΔF/F. Hearts were stimulated with 150 ms cycle length followed by premature stimulation of 10 beats of S2 until refractoriness or VT induction. Propagation and duration of Ca^2+^ transients were mapped using (d*F*/d*t*)_max_ and 75% recovery, respectively, as previously described (Kim et al., 2015).

### Statistics

Statistical analysis of Ca^2+^ imaging and biochemical data was performed using Origin 8 (OriginLab). Data are presented as mean ± standard error (SEM) for single cell and ± standard deviation (SD) for intact heart optical mapping. Uppercase n (*N*) = number of animals, lowercase *n* = number of VMs. Statistical significance between groups were performed using Student’s *t*-test (paired and unpaired), Fisher’s exact test and one-way ANOVA with Bonferroni *post hoc* test where appropriate. For all analyses, a *p*-value of less than 0.05 was considered significant.

**FIGURE 1:**
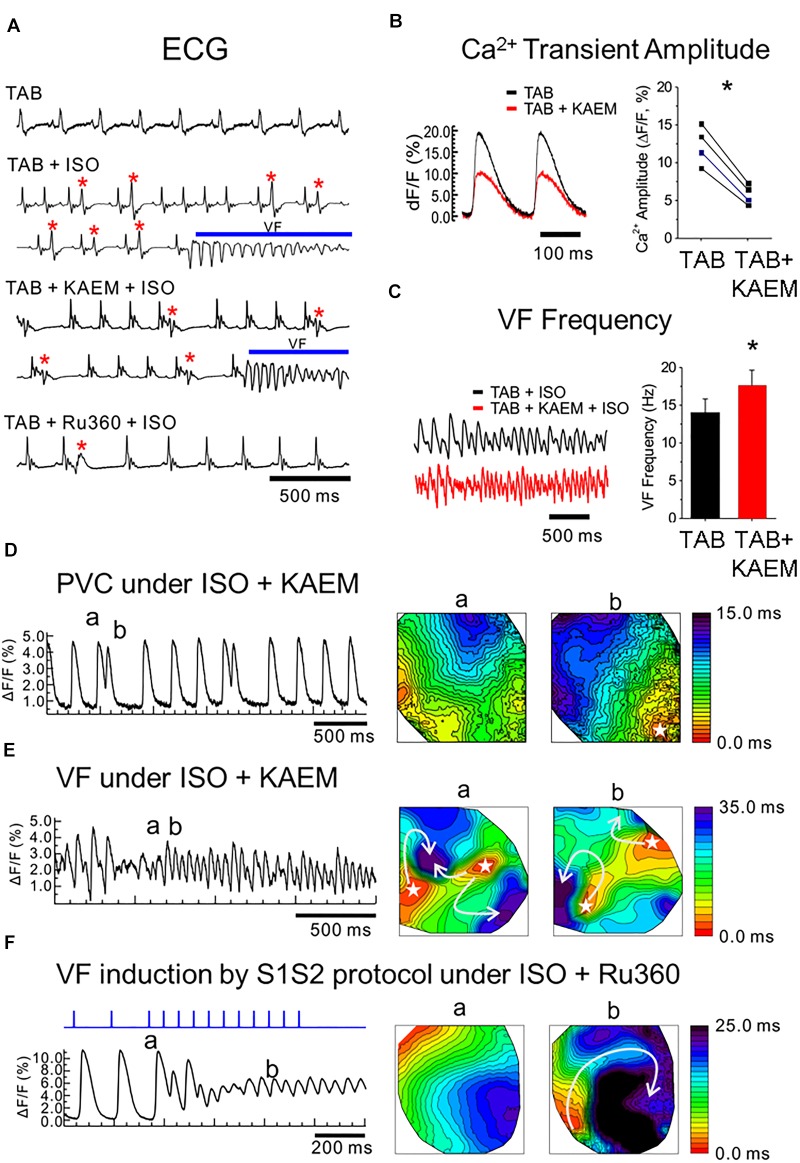
MCU enhancer kaempferol exacerbates ventricular arrhythmias in TAB rat hearts. **(A)** ECG recordings from *ex vivo* intact hearts. *TAB*: ECGs under normal condition. *TAB+ISO*: ISO (50 nmol/L) perfusion frequently induces PVCs (red stars) and VT/VF in TAB rat hearts. *TAB+KAEM+ISO*: Pretreatment of kaempferol (KAEM, 1 μmol/L) failed to prevent PVCs and VT/VF induction. *TAB+RU360+ISO*: Ru360 pretreatment (2 μmol/L) reduced PVC events (red star) and prevented VT/VF induction under ISO in TAB rat hearts. **(B)** KAEM reduced Ca^2+^ transient amplitude (12.3 ± 2.6 Δ*F*/*F* before KAEM vs. 5.8 ± 1.3 Δ*F*/*F* after KAEM, ^∗^*p* = 0.0018, paired Student’s *t*-test, *N* = 4). **(C)** KAEM increases VF frequency (14.1 ± 1.8 Hz before KAEM vs. 17.6 ± 2.1 Hz after KAEM). **(D,E)** Frequent PVCs before VF induction and focal activity in the presence of KAEM. Activation maps are shown in the right panel. **(F)** Conduction block and reentry formation by S1S2 pacing protocol in the presence of Ru360 (*N* = 4).

## Results

### Mitochondrial Ca^2+^ Accumulation Exacerbates the Proarrhythmic Phenotype of TAB Hearts *ex vivo*

We have previously reported that the rat model hypertrophy induced by TAB is highly arrhythmogenic, with incidences of non-sustained ventricular tachycardia and fibrillation (VT/VF) occurring in 100% of TAB hearts exposed to 50 nmol/L ISO (Kim et al., 2017). We investigated whether the MCU activator, kaempferol (Montero et al., 2004; Vay et al., 2007), increases mitochondrial Ca^2+^ accumulation to reduce cytosolic Ca^2+^ and suppress ventricular arrhythmias in this model. Hearts were isolated and perfused retrogradely as described in “Materials and Methods” and echocardiograms (ECGs) were monitored to investigate arrhythmogenesis under kaempferol and Ru360. ISO induced frequent premature ventricular contractions (PVCs) leading to VFs in TAB rat hearts (Figure 1A ECG traces under **TAB+ISO**). Pretreatment with MCU activator, kaempferol (10 μmol/L), did not prevent PVCs and VF induction. ECG traces in Figure 1A (**TAB+KAEM+ISO** panel) shows frequent PVCs (red stars) that led to VFs (blue bar) under kaempferol. However, MCU blocker Ru360 (2 μmol/L) suppressed number of PVCs and prevented VFs in TAB rat hearts (Figure 1A, **TAB+Ru360+ISO** panel).

We investigated the effect of kaempferol on Ca^2+^ handling and arrhythmogenesis using optical mapping. Kaempferol reduces the Ca^2+^ transient amplitude by 53% (Figure 1B, ^∗^*p* = 0.002) but despite smaller Ca^2+^ transients, kaempferol induced transient VTs in two of four TAB hearts, even without ISO. Addition of ISO caused frequent PVCs (Figure 1D) and long-lasting VFs (Figure 1E) in three of four hearts. Activation maps of PVCs (Figure 1D) and VFs (Figure 1E) suggest that focal activity play a major role in the initiation and maintenance of VFs. The frequencies of VF were significantly higher with kaempferol (17.6 ± 2.1 Hz, *n* = 3, in kaempferol vs. 14 ± 1.8 Hz, *n* = 7, control TAB with ISO, ^∗^*p* = 0.042, Figure 1C). In contrast, MCU inhibitor, Ru360 (Matlib et al., 1998; García-Rivas Gde et al., 2006), suppressed spontaneous VT/VFs in the presence of ISO in four of four hearts. S1S2 pacing induced reentry and VFs in two of four hearts in the presence of Ru360 (Figure 1F), suggesting that conduction block, not focal activity, underlies VF induction during S1S2 pacing in Ru360 group. These intact heart optical mapping data suggest that enhancement of mitochondrial Ca^2+^ accumulation may exacerbate ventricular arrhythmias in TAB rat hearts through increasing focal activity.

### Pharmacological Enhancers of Mitochondrial Ca^2+^ Accumulation Modulate Time Course, Not Amplitude During Periodic Pacing

To gain mechanistic insights as to how increasing mitochondrial Ca^2+^ accumulation affects global Ca^2+^ handling in VMs, we used a genetically encoded fluorescent Ca^2+^ biosensor, mtRCamp1h. The RCamp1h indicator, with a *K*d ∼ 1.3 μM (Akerboom et al., 2013), was fused with an N-terminal cytochrome C oxidase subunit IV tag for targeting to the mitochondrial matrix. We generated adenovirus encoding the sensor, and VMs isolated from control rat hearts were infected with adenovirus at a MOI of 10 and cultured for 48 h prior to experimentation. Rat myocytes are thought to preserve electrical properties and structure including T-tubule organization for the first 48 h of culture (Banyasz et al., 2008).

**FIGURE 2:**
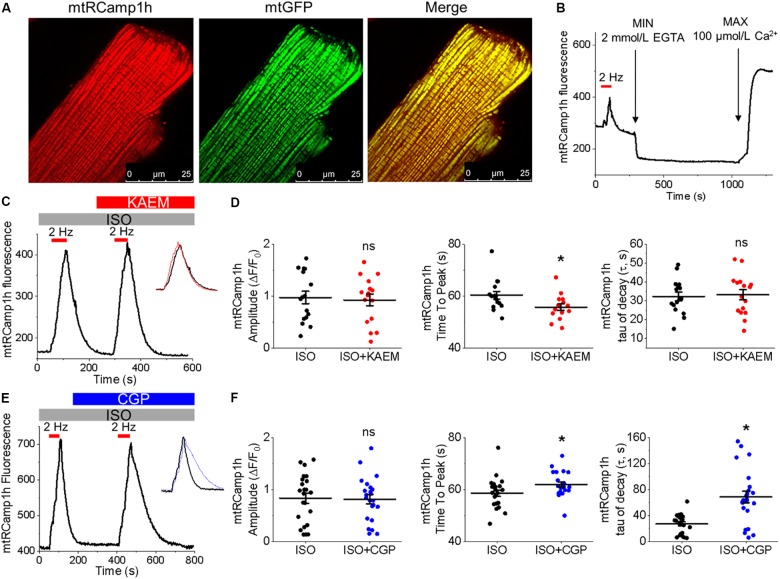
Expression of biosensor mtRCamp1h in cultured control rat VMs reveals mitochondrial Ca^2+^ accumulation is modified by kaempferol and CGP-37157. **(A)** Representative images of a cultured VM 48 h after adenoviral infection with mtRCamp1h (left, red, MOI of 10) and mitochondrial-targeted GFP (center, green, MOI of 10). A merged image is indicated on the right. **(B)** Recording of a permeabilized VM infected with mtRCamp1h. *F*max and *F*min were assessed by addition of EGTA (2 mmol/L) and Ca^2+^ (100 μmol/L), respectively. **(C)** Representative trace from cultured VM expressing mtRCamp1h. VMs were treated with ISO (50 nmol/L) and paced at 2 Hz for 60 s intervals (red bars), then treated with kaempferol (KAEM, 10 μmol/L) and paced again peaks are overlaid in the inset for visualization, with ISO in black, ISO+KAEM in red. Graphs in **(D)** depict mean data ± SEM of mtRCamp1h transient amplitude, time to transient peak and tau of decay (*n* = 16 per group, *N* = 5, ^∗^*p* < 0.05, paired Student’s *t*-test). **(E)** Representative trace from VM expressing mtRCamp1h. VMs were treated with ISO and paced at 2 Hz for 60 s intervals (red bars), then treated with CGP-37157 (CGP, 1 μmol/L) and paced again. Peaks are overlaid in the inset for visualization, with ISO in black, ISO+CGP in blue. Graphs in **(F)** depict mean data ± SEM from mtRCamp1h transient amplitude, time to transient peak and tau of decay (*n* = 23, *N* = 4, ^∗^*p* < 0.05, paired Student’s *t*-test).

**FIGURE 3:**
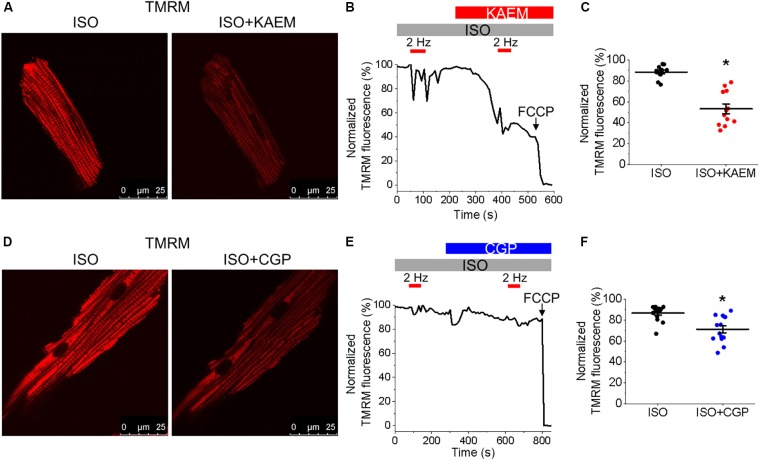
Pharmacological enhancers of mitochondrial Ca^2+^ accumulation modulate Δψm in cultured control rat VMs. **(A)** Representative images of a VM treated with ISO (50 nmol/L) before and after application of kaempferol (KAEM, 10 μmol/L). Δψm of VMs was monitored by TMRM fluorescence (20 μmol/L). **(B)** Representative recording of changes in Δψm in response to application of ISO and pacing at 2 Hz (red bars) for 60 s, followed by application of KAEM. TMRM signal was normalized to minimum fluorescence obtained by application of FCCP (50 μmol/L) and represented as percentage of baseline. Graph in **(C)** depicts pooled data for minimum fluorescence after pacing and application of ISO or KAEM (*n* = 12, *N* = 4, ^∗^*p* < 0.05, paired Student’s *t*-test). **(D)** Representative images of a VM treated with ISO before and after application of CGP-37157 (CGP, 1 μmol/L). Δψm of VMs was monitored by TMRM fluorescence. **(E)** Representative recordings of changes in Δψm in response to application of ISO and pacing at 2 Hz for 60 s, followed by application of CGP. Graph in **(F)** depicts pooled mean data ± SEM for minimum fluorescence after pacing and application of ISO or KAEM (*n* = 14, *N* = 3, ^∗^*p* < 0.05, paired Student’s *t*-test).

The correct cellular localization of mtRCamp1h was confirmed with co-expression of mitochondrial matrix-targeted GFP, as shown in Figure 2A. As shown in Figure 2B, basal mtRCamp1h fluorescence appears well within the dynamic range of the probe when adenovirally expressed in VMs. Furthermore, as seen in Figure 2B, we can indeed observe measurable changes in mitochondrial Ca^2+^ concentration, as indicated by an increase in mtRCamp1h fluorescence, when cultured control VMs are paced at 2 Hz for 1 min under β-adrenergic stimulation with ISO (pacing indicated by red bar). We did not observe significant loading of mitochondria with Ca^2+^ under baseline conditions with no ISO stimulation (data not shown). To determine *F*min and *F*max for mtRCamp1h, VMs were saponin-permeabilized (0.001%) and equilibrated with a solution containing thapsigargin (10 μmol/L) to deplete SR Ca^2+^, cytochalasin D (10 μmol/L) to reduce cell contraction, as well as FCCP (20 μmol/L) and ionomycin (5 μmol/L) to dissipate mitochondrial membrane potential (Δψm). Solution containing Ca^2+^ buffer EGTA at high concentration (2 mmol/L) was applied to obtain minimum mtRCamp1h fluorescence, while maximum fluorescence was achieved by application of Ca^2+^ (100 μmol/L), as illustrated in Figure 2B.

Measurement of peak mtRCamp1h signal indicated accumulation of [Ca^2+^]_m_ during workload, but after treatment with MCU enhancer kaempferol (10 μmol/L; Figure 2C) or NCLX blocker CGP-37157 (1 μmol/L; Figure 2E), no significant change in transient amplitude (an increase in [Ca^2+^]_m_) was observed (Figures 2D,F, respectively). However, accumulation of mitochondrial Ca^2+^ during pacing was significantly faster after treatment with kaempferol (Figure 2D, time to peak 59.31 ± 1.01 s. ISO vs. 55.84 ± 1.24 s. ISO and kaempferol, ^∗^*p* = 0.03), while the time constant of transient decay, τ, was significantly increased after application of CGP-37157 (Figure 2F, τ = 27.16 ± 3.10 ISO vs. 68.70 ± 8.81 ISO and CGP-37157, ^∗^*p* < 0.001). The time to peak of transient after application of CGP-37157 was also increased (58.59 ± 1.23 s. ISO vs. 62.01 ± 0.98 s. ISO and CGP-37157, ^∗^*p* = 0.01). This indicates that while pharmacological enhancers of [Ca^2+^]_m_ do not increase overall mitochondrial Ca^2+^ loading in cultured control VMs during workload of 1 min pacing, they modify the time course for which those VMs accumulate or retain Ca^2+^. The finding that the rate of mitochondrial Ca^2+^ uptake in intact myocytes is only modestly accelerated with kaempferol and even slowed down with CGP-37157 suggest the existence of overriding feedback mechanisms to prevent mitochondrial Ca^2+^ overload that can cause cell death (Broekemeier et al., 1998; Hüser and Blatter, 1999; Elrod et al., 2010).

### Enhanced Mitochondrial Ca^2+^ Accumulation Dissipates Δψm

It was established that the excessive mitochondrial Ca^2+^ uptake can be effectively limited by a reduction in Δψm at least in part via activation of mitochondrial Ca^2+^-dependent K^+^ channels (O’Rourke et al., 2005; Stowe et al., 2006). While irreversible mPTP opening collapses Δψm, brief openings of the pore may also provide a protective efflux mechanism against mitochondrial Ca^2+^ overload (Broekemeier et al., 1998; Elrod et al., 2010). We used isolated control rat VMs stained with voltage sensitive dye TMRM (20 μmol/L for 1 min) to determine if enhancement of mitochondrial Ca^2+^ accumulation modifies Δψm (Figure 3). As in Figure 2, VMs under β-adrenergic stimulation with ISO were paced at 2 Hz for 1 min, as indicated by red bars. Representative images Figure 3A shows VMs before and after the application of kaempferol. A representative trace is shown in Figure 3B, whereby signal was normalized to minimum fluorescence obtained by the application of FCCP (50 μmol/L). Application of kaempferol significantly reduced TMRM fluorescence (Figure 3C, 88.31 ± 1.73% ISO vs. 53.17 ± 4.69% ISO and kaempferol, ^∗^*p* < 0.001). Application of CGP-37157 had similar effects (Figures 3D–F, 86.49 ± 1.95% ISO vs. 71.15 ± 3.38% ISO and CGP-37157, ^∗^*p* < 0.001, respectively). The decrease in driving force due to the drop in Δψm may explain why kaempferol or CGP-37157 are not able to increase the amplitude of [Ca^2+^]_m_ effectively limiting mitochondrial Ca^2+^ uptake, as measured with mtRCamp1h in Figure 2.

**FIGURE 4:**
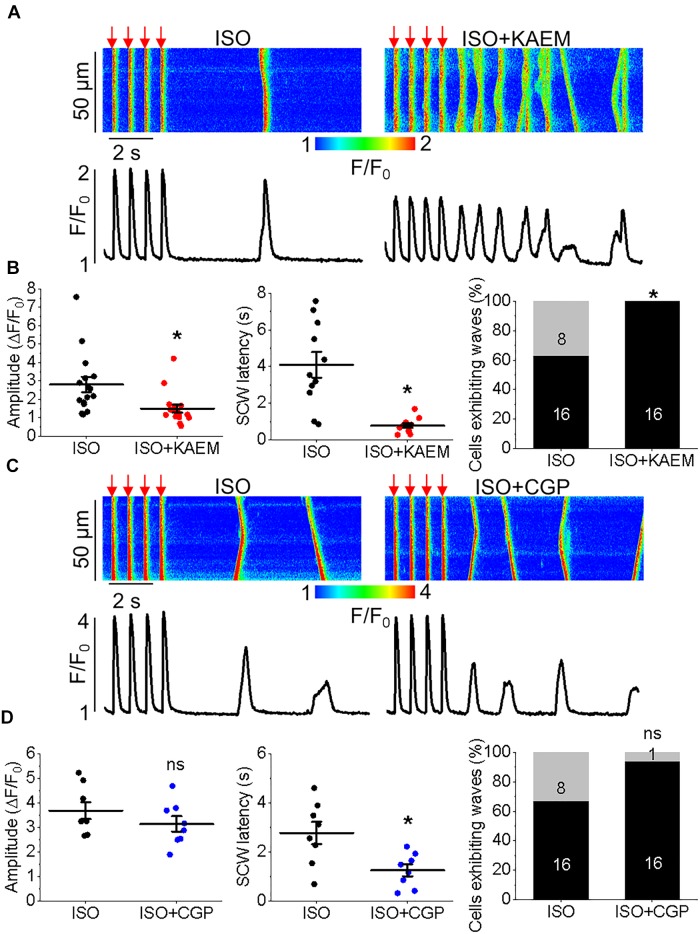
Enhanced mitochondrial Ca^2+^ accumulation promotes proarrhythmic spontaneous Ca^2+^ waves in control cultured rat VMs. **(A)** Representative confocal line scan images of Ca^2+^ transients and Rhod-2 fluorescence (*F*/*F*_0_) profiles ISO treated (50 nmol/L) rat VM undergoing 2 Hz pace-pause protocol (red arrows) to induce SCW, before and after application of kaempferol (KAEM, 10 μmol/L). Graphs in **(B)** depict mean data ± SEM from Ca^2+^ transient amplitude (*n* = 15, *N* = 5, ^∗^*p* < 0.05, paired Student’s *t*-test), SCW latency (*n* = 11, *N* = 5, paired Student’s *t*-test) and the percentage of cells exhibiting waves (*N* = 5, ^∗^*p* < 0.05, Fisher’s exact test). **(C)** Representative confocal line scan images of Ca^2+^ transients and Rhod-2 fluorescence (*F*/*F*_0_) profiles of ISO treated rat VM undergoing 2 Hz pace-pause protocol before and after application of CGP-37157 (CGP, 1 μmol/L). Graphs in **(D)** depict mean data ± SEM from Ca^2+^ transient amplitude (*n* = 7, *N* = 4, ns is not significant, paired Student’s *t*-test), SCW latency (*n* = 8, *N* = 4, ^∗^*p* < 0.05, paired Student’s *t*-test) and the percentage of cells exhibiting waves (*N* = 4, ns is not significant, Fisher’s exact test).

### Facilitation of Mitochondrial Ca^2+^ Accumulation Promotes Proarrhythmic SCWs in VMs

Having demonstrated the effects of kaempferol and CGP-37157 on mitochondrial Ca^2+^ and Δψm, we next sought to establish the effects of modulating mitochondrial Ca^2+^ on cytosolic Ca^2+^ handling in VMs, as illustrated in Figure 4. Cultured control VMs were loaded with the fluorescent Ca^2+^ indicator Rhod-2 and we recorded cytosolic Ca^2+^ in the presence of β-adrenergic receptor agonist ISO (50 nmol/L), subjected to a burst-pace pause protocol (2 Hz, 20 s). We assessed Ca^2+^ transient amplitude and SCW latency as an indication of the propensity for arrhythmogenic Ca^2+^ release. As shown in Figures 4A,B, VMs treated with ISO and kaempferol have a significantly lower Ca^2+^ transient amplitude in comparison to ISO alone (2.77 ± 0.44 Δ*F*/*F*_0_ ISO vs. 1.57 ± 0.23 Δ*F*/*F*_0_ ISO and kaempferol, ^∗^*p* = 0.006). We observed a fivefold shortening in SCW latency after treatment with kaempferol (^∗^*p* < 0.001), as well a significant increase in the percentage of VMs exhibiting SCWs (63% ISO vs. 100% ISO and kaempferol, ^∗^*p* < 0.001). A similar pattern was obtained in VMs treated with CGP-37157 (Figures 4C,D), whereby the SCW latency was shortened approximately twofold (^∗^*p* = 0.002). Changes in Ca^2+^ transient amplitude and the percentage of cells exhibiting waves were not significant on treatment with CGP-37157.

**FIGURE 5:**
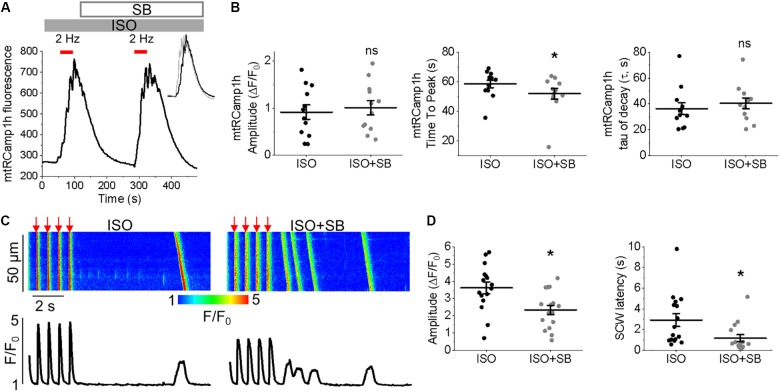
MCU enhancer SB 202190 has similar effects to kaempferol in cultured control rat VMs. **(A)** Representative trace from cultured VM expressing mtRCamp1h. VMs were treated with ISO (50 nmol/L) and paced at 2 Hz for 60 s intervals (red bars), then treated with SB 202190 (SB) and paced again. Peaks are overlaid in the inset for visualization, with ISO in black, ISO+SB in gray. Graphs in **(B)** depict mean data ± SEM of mtRCamp1h transient amplitude, time to transient peak and tau of decay (*n* = 12 per group, *N* = 3, ^∗^*p* < 0.05, paired Student’s *t*-test). **(C)** Representative confocal line scan images of Ca^2+^ transients and Rhod-2 fluorescence (*F*/*F*_0_) profiles ISO treated (50 nmol/L) rat VM undergoing 2 Hz pace-pause protocol (red arrows) to induce SCW, before and after application of SB 202190 (SB, 30 μmol/L). Graphs in **(D)** depict mean data ± SEM from Ca^2+^ transient amplitude (*n* = 16, *N* = 3, ^∗^*p* < 0.05, paired Student’s *t*-test) and SCW latency (*n* = 16, *N* = 3, ^∗^*p* < 0.05, paired Student’s *t*-test).

**FIGURE 6:**
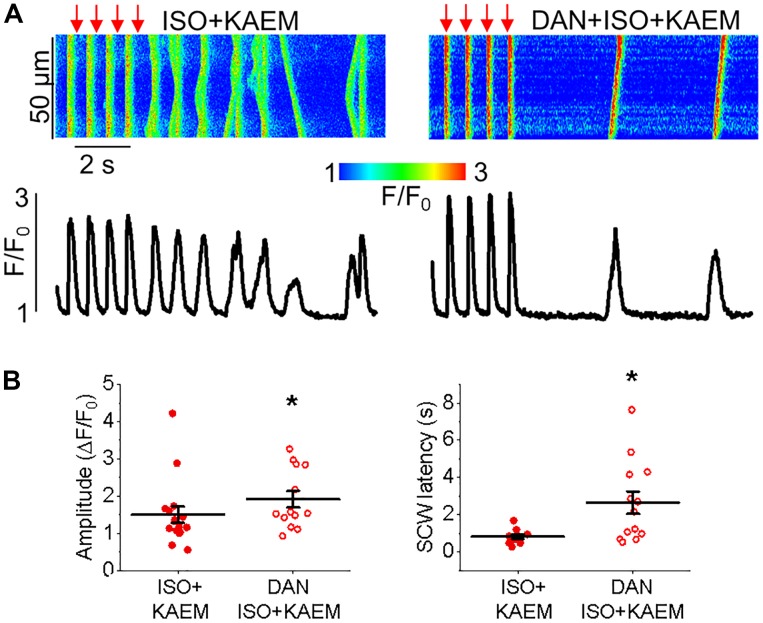
Inhibition of RyR with dantrolene reduces proarrhythmic effects of kaempferol on Ca^2+^ handling in cultured control rat VMs. **(A)** Representative confocal line scan images of Ca^2+^ transients and Rhod-2 fluorescence (*F*/*F*_0_) profiles of TAB rat VMs undergoing 2 Hz pace-pause protocol (red arrows) to induce SCWs. VMs were treated with isoproterenol (ISO, 50 nmol/L) and kaempferol (KAEM, 10 μmol/L), or were pretreated for 5 min with dantrolene (DAN, 2 μmol/L) before ISO and KAEM treatment. Graphs in **(B)** depict mean data ± SEM from Ca^2+^ transient amplitude (*n* = 13–15, *N* = 3–4, ^∗^*p* < 0.05, Student’s *t*-test) and SCW latency (*n* = 11–13, *N* = 3–4, ^∗^*p* < 0.05, Student’s *t*-test).

**FIGURE 7:**
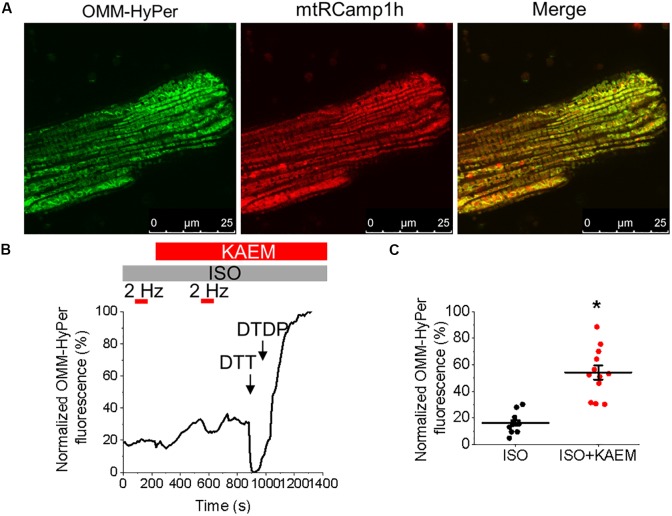
Pharmacological enhancement of mitochondrial Ca^2+^ accumulation increases mitochondrial ROS in cultured control rat VMs. **(A)** Representative image of a cultured control VM 48 h after adenoviral infection with biosensor OMM-HyPer (left, green, MOI of 10) and mtRCamp1h (center, red, MOI of 10). A merged image is indicated on the right. **(B)** Representative trace of OMM-HyPer fluorescence from a VM treated with ISO (50 nmol/L), paced at 2 Hz (red bars) and subsequently treated with kaempferol (KAEM, 10 μmol/L) before further pacing. OMM-HyPer signal was normalized to minimum fluorescence obtained by application of dithiothreitol (DTT, 5 mmol/L) and maximum fluorescence obtained by application of deoxythymidine diphosphate (DTDP, 200 μmol/L). Graph in **(C)** depicts mean data ± SEM for maximum normalized fluorescence after pacing and application of ISO and KAEM (*n* = 12, *N* = 4, ^∗^*p* < 0.05, paired Student’s *t*-test).

To provide additional evidence that facilitation of mitochondrial Ca^2+^ accumulation exerts strong deleterious effects on intracellular Ca^2+^ handling we performed experiments using another MCU enhancer SB 202190 (Montero et al., 2004) (Figure 5). As seen from experiments in ISO-treated VMs expressing mtRCamp1h, application of SB 202190 (30 μmol/L) moderately but significantly accelerated rate of mitochondrial Ca^2+^ uptake while failed to increase overall amplitude of mitochondrial Ca^2+^ transients (Figures 5A,B), in line with the effects of kaempferol (Figure 2C). Importantly, similar to kaempferol, incubation with SB reduced amplitude of Ca^2+^ transients and shortened and latency of SCWs in periodically paced ISO-treated cells (Figures 5C,D).

Enhanced propensity to generate SCWs is often attributed to abnormally high activity of SR Ca^2+^ release channels, RyRs (Bers, 2002). To test whether stabilization of RyRs can attenuate kaempferol–mediated effects on Ca^2+^ handling, we treated VMs with dantrolene (2 μmol/L, 5 min), a specific inhibitor of RyR. Figure 6 shows that dantrolene restores Ca^2+^ transient amplitude and significantly reduces kaempferol-mediated shortening of SCW latency in ISO-treated VMs (SCW latency 3.34 ± 0.73 s ISO and kaempferol vs. 2.64 ± 0.60 s ISO, kaempferol and dantrolene, ^∗^*p* < 0.001).

Taken together, these data suggest that facilitation of mitochondrial Ca^2+^ accumulation promotes generation of proarrhythmic SCWs by enhancement of activity of RyRs.

**FIGURE 8:**
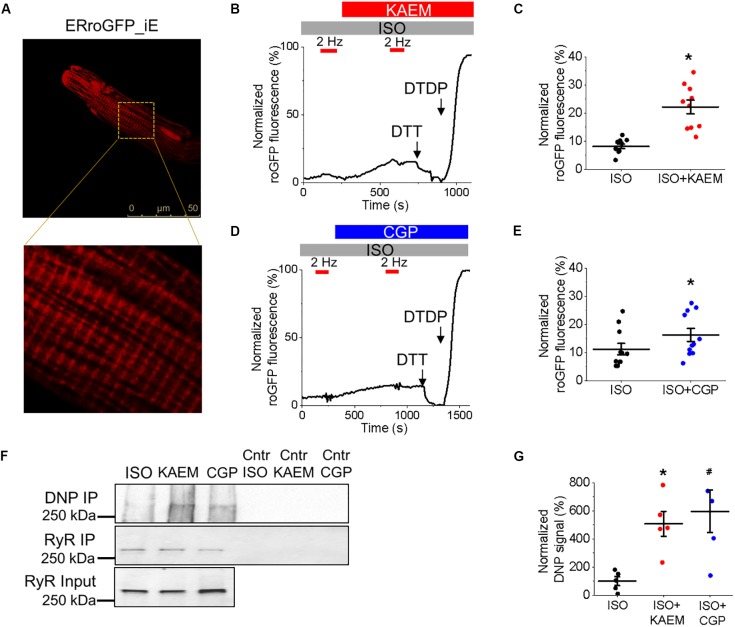
Pharmacological enhancement of mitochondrial Ca^2+^ accumulation increases ROS in the vicinity of RyR in cultured control rat VMs. **(A)** Representative image of a cultured control VM 48 h after adenoviral infection with biosensor ERroGFP_iE (MOI of 10). **(B)** Representative trace of ERroGFP_iE fluorescence from a VM treated with ISO (50 nmol/L), paced at 2 Hz (red bars) and subsequently treated with kaempferol (KAEM, 10 μmol/L) before further pacing. ERroGFP_iE signal was normalized to minimum fluorescence obtained by application of dithiothreitol (DTT, 5 mmol/L) and maximum fluorescence obtained by application of deoxythymidine diphosphate (DTDP, 200 μmol/L). Graph in **(C)** depicts mean data ± SEM for maximum normalized fluorescence after pacing and application of ISO and KAEM (*n* = 10, *N* = 3, ^∗^*p* < 0.05, paired Student’s *t*-test). **(D)** Representative trace of ERroGFP_iE fluorescence from a VM treated with ISO, paced at 2 Hz and subsequently treated with CGP-37157 (CGP, 1 μmol/L) before further pacing. ERroGFP_iE signal was normalized to minimum fluorescence obtained by application of DTT and maximum fluorescence obtained by application of DTDP. Graph in **(E)** depicts mean data ± SEM for maximum fluorescence after pacing and application of ISO and CGP (*n* = 11, *N* = 3, ^∗^*p* < 0.05, paired Student’s *t*-test). **(F)** RyR in freshly isolated control VMs was immunoprecipitated (IP) and immunoblotted for oxidation using DNP antibody. Representative images of DNP IP, RyR IP and RyR input signal from VMs treated with ISO, ISO and KAEM, or ISO and CGP, with controls (Cntr). **(G)** Quantification of normalized DNP signal (%, *N* = 5, ^∗^*p* and ^#^*p* < 0.05 vs. ISO, one-way ANOVA with Bonferroni *post hoc* test).

### Kaempferol and CGP-37157 Increase ROS and Oxidation of RyRs

RyR is well established as a ROS sensor, with increased oxidation and thus RyR activity associated with enhanced propensity for spontaneous SR Ca^2+^ release and proarrhythmic SCWs. Mitochondria is a major intracellular source of ROS and excessive RyR oxidation by mitochondria-derived ROS has been demonstrated in many models of HF and aging (Zima and Blatter, 2006). Facilitation of mitochondrial Ca^2+^ uptake may accelerate the rate of emission of ROS by stimulating electron transport (Bertero and Maack, 2018). Therefore, to test the possible effects of kaempferol on mitochondrial redox state, control VMs were infected (MOI of 10) with adenovirus construct carrying sequence encoding the novel H_2_O_2_ probe OMM-HyPer, and cultured for 48 h prior to imaging. As shown in Figure 7, application of kaempferol significantly increases the signal of mitochondrial-targeted peroxide-sensitive indicator (normalized fluorescence 16.29 ± 2.11% ISO vs. 54.23 ± 5.31% ISO and kaempferol, ^∗^*p* < 0.001), confirming that facilitation of mitochondrial Ca^2+^ accumulation induces mitochondrial ROS release.

Considering the close proximity of mitochondria and SR, we next sought to determine whether facilitation of mitochondrial Ca^2+^ accumulation leads to an increase in local ROS levels in the vicinity of RyR in cultured control VMs, measured using the ER-tuned redox-sensitive biosensor ERroGFP_iE (Avezov et al., 2013). The GFP sensor contains engineered cysteine residues that enable formation of di-thiol in response to oxidant stress (Cannon and Remington, 2009). After we generated adenovirus encoding the sensor, VMs were infected at a MOI of 10 and cultured for 48 h prior to imaging. As shown in Figure 8A, ERroGFP_iE-infected VMs exhibit a striated pattern indicative of SR targeting of the probe.

Figures 8B,D show representative recordings where signal of ERroGFP_iE was normalized to minimal fluorescence obtained by application of reducing agent dithiothreitol (DTT, 5 mmol/L) and maximal fluorescence obtained by application of oxidizing agent 2,2’-dithiodipyridine (DTDP, 200 μmol/L). At baseline under β-adrenergic stimulation with ISO, little change in oxidation in the form of increased fluorescence and di-thiol formation is observed. However, treatment with kaempferol or CGP-37157 still leads to a measurable and significant increase in ERroGFP_iE signal (8.19 ± 0.83% ISO vs. 22.20 ± 2.49% ISO and kaempferol, ^∗^*p* < 0.001 and 11.21 ± 2.03% ISO vs. 16.26 ± 2.32 ISO and CGP-37157, ^∗^*p* = 0.006, respectively), indicative of increased oxidation and ROS emission in close proximity of RyR (Figures 8C,E, respectively).

To directly test the hypothesis that pharmacological enhancement of [Ca^2+^]_m_ accumulation results in increased RyR oxidation, the free thiol content of immunoprecipitated RyRs was measured using DNP-antibody. Figure 8F demonstrates that treatment with kaempferol or CGP-37157 (for 5 and 10 min, respectively) significantly increases oxidation of RyR fivefold and sixfold, respectively (Figure 8G, ^∗^*p* = 0.047 and ^#^*p* = 0.015), shown by increased DNP signal. These data demonstrate that in control VMs, enhanced mitochondrial Ca^2+^ accumulation leads to increased ROS in the closely situated SR. Subsequent increased RyR oxidation is likely the responsible mechanism for the shortened latency for proarrhythmic RyR-mediated SCWs in VMs with pharmacologically enhanced mitochondrial Ca^2+^ accumulation.

### The Effects of Pharmacological Modulators on Mitochondrial Ca^2+^ Accumulation in TAB VMs

Kaempferol and CGP-37157 modulate mitochondrial Ca^2+^ accumulation, membrane potential and ROS emission in cultured VMs from control rat hearts, as well as intracellular Ca^2+^ cycling and SCW generation. However, in the diseased heart, redox balance is altered and both intracellular and mitochondrial Ca^2+^ handling can be compromised already (Kim et al., 2017).

**FIGURE 9:**
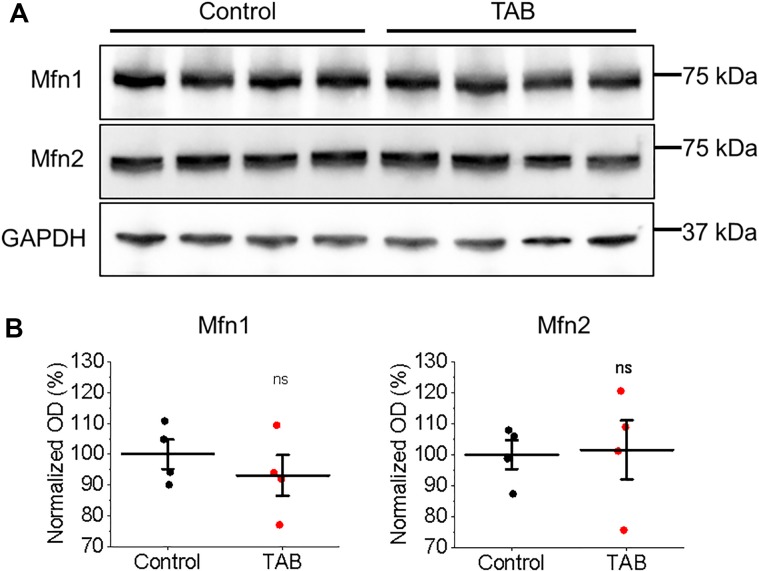
Expression of mitochondrial associated membrane proteins Mfn1 and Mfn2 is not altered in hypertrophic rat VMs. **(A)** Representative images of control and TAB VMs immunoblotted for expression of mitofusin 1 (Mfn1) and mitofusin 2 (Mfn2). GAPDH was used as a loading control. **(B)** Quantification of normalized optical densities (*N* = 4 per group, Student’s *t*-test).

**FIGURE 10:**
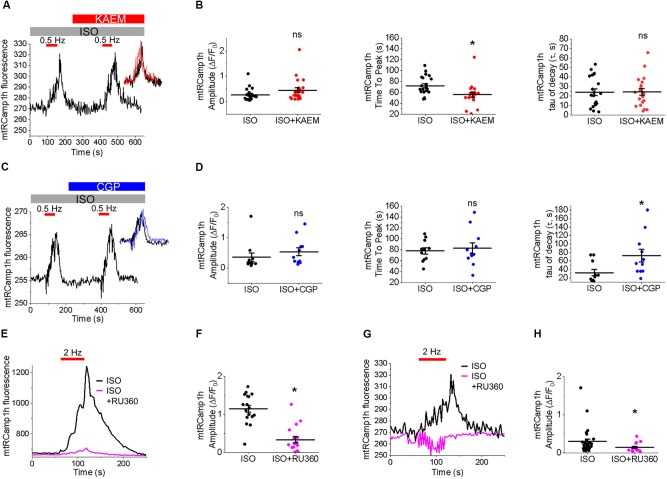
Pharmacological modulators of mitochondrial Ca^2+^ in TAB VMs exert similar effects to those on controls, despite hypertrophy-related impairment in mitochondrial Ca^2+^ homeostasis. **(A)** Representative trace from cultured TAB VM expressing mtRCamp1h. TAB VMs were treated with ISO (50 nmol/L) and paced at 0.5 Hz for 60 s intervals (red bars), before treatment with kaempferol (KAEM, 10 μmol/L) and further pacing. Peaks are overlaid in the inset for visualization, with ISO in black, ISO+KAEM in red. Graphs in **(B)** depict mean data ± SEM of mtRCamp1h transient amplitude, time to transient peak and tau of decay (*n* = 22 per group, *N* = 4, ^∗^*p* < 0.05, paired Student’s *t*-test). **(C)** Representative trace from cultured TAB VM expressing mtRCamp1h. VMs were treated with ISO and paced at 0.5 Hz for 60 s intervals (red bars), before treatment with CGP-37157 (CGP, 1 μmol/L) and further pacing. Peaks are overlaid in the inset for visualization, with ISO in black, ISO+CGP in blue. Graphs in **(D)** depict mean data ± SEM from mtRCamp1h transient amplitude, time to transient peak and tau of decay (*n* = 11, *N* = 3, ^∗^*p* < 0.05, paired Student’s *t*-test). **(E)** Representative trace from a cultured control VM expressing mtRCamp1h. Myocytes were treated with ISO (black line) and paced at 2 Hz for 60 s (red bar), or pretreated with Ru360 (2 μmol/L) for 30 min before treatment with ISO and pacing at 2 Hz (pink line). Graph in **(F)** depicts mean data ± SEM from mtRCamp1h transient amplitude (*n* = 17–18, *N* = 3, ^∗^*p* < 0.05, Student’s *t*-test). **(G)** Representative trace from a cultured TAB VM expressing mtRCamp1h. Myocytes were treated with ISO (black line) and paced at 2 Hz for 60 s (red bar), or pretreated with Ru360 (2 μmol/L) for 30 min before treatment with ISO and pacing at 0.5 Hz (pink line). Graph in **(H)** depicts mean data ± SEM from mtRCamp1h transient amplitude (*n* = 17, *N* = 4, ^∗^*p* < 0.05, Student’s *t*-test).

To test whether tethering of mitochondria to the SR is altered in TABs rat VMs, we performed western blot analysis of expression levels of mitofusin 1 and mitofusin 2; proteins that scaffold these two organelles (de Brito and Scorrano, 2008; Chen et al., 2012; Filadi et al., 2015). We did not find changes in expression levels of both these proteins in VMs of TABs vs. those of controls (Figure 9). Next, isolated TAB VMs were infected with adenoviruses to express mitochondrial Ca^2+^ indicator mtRCamp1h. Figures 10A–D demonstrate that the effects of kaempferol and CGP-37157 on mitochondrial Ca^2+^ accumulation are qualitatively similar to those in control VMs. As in controls, kaempferol accelerates Ca^2+^ accumulation (time to peak 72.43 ± 3.75 s ISO vs. 56.14 ± 4.47 s ISO and kaempferol, ^∗^*p* < 0.001) but the amplitude does not change significantly, while CGP-37157 impedes mitochondrial Ca^2+^ transient decay (tau of decay 32.08 ± 7.49 s ISO vs. 72.38 ± 14.99 s ISO and CGP-37157, ^∗^*p* = 0.006). In addition, we tested the effects of MCU inhibitor Ru360. Figures 10E,F show representative traces and respective pooled data for mitochondrial Ca^2+^ transients in ISO-treated control VMs under basal conditions and after 30 min incubation with 2 μmol/L Ru360. Figures 10G,H demonstrates that Ru360 reduces mitochondrial Ca^2+^ accumulation in TABs (0.29 ± 0.07 Δ*F*/*F*_0_ ISO vs. 0.14 ± 0.03 Δ*F*/*F*_0_ ISO and Ru360, ^∗^*p* < 0.001). Notably, the amplitude of pacing-induced mitochondrial Ca^2+^ transients in ISO-treated TAB VMs is significantly smaller that in controls (amplitude 1.15 ± 0.09 Δ*F*/*F*_0_ ISO Control vs. 0.30 ± 0.07 Δ*F*/*F*_0_ ISO TAB, ^∗^*p* < 0.001, Student’s *t*-test). Given this difference, we next performed experiments using freshly isolated VMs from hypertrophic TAB rat hearts, to test whether kaempferol and CGP-37157 have deleterious effects in a disease setting, or on the contrary could be protective.

### Enhanced Mitochondrial Ca^2+^ Accumulation Further Perturbs Intracellular Ca^2+^ Cycling in TAB VMs

**FIGURE 11:**
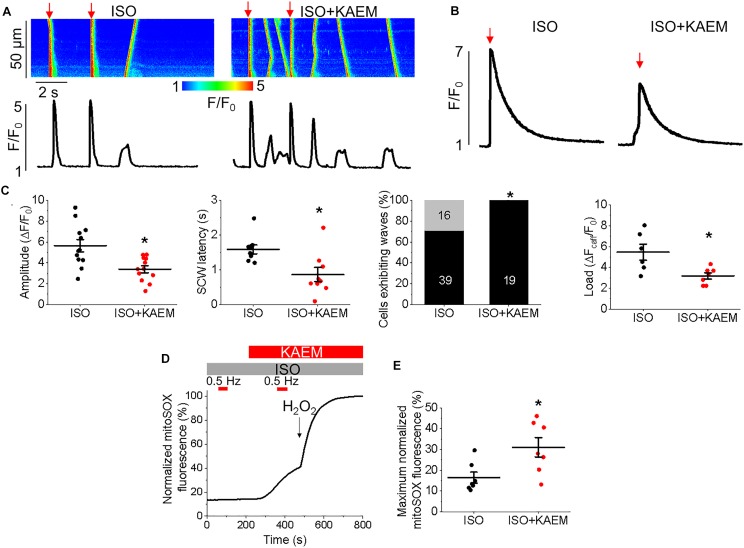
Enhanced mitochondrial Ca^2+^ accumulation by kaempferol promotes proarrhythmic SCWs in TAB rat VMs. **(A)** Representative confocal line scan images of Ca^2+^ transients and Rhod-2 fluorescence (*F*/*F*_0_) profiles of ISO treated (50 nmol/L) TAB rat VMs undergoing 0.5 Hz pace-pause protocol (red arrows) to induce SCWs, before and after application of kaempferol (KAEM, 10 μmol/L). **(B)** Representative traces of caffeine-induced Ca^2+^ transients (10 mmol/L). Graphs in **C** depict mean data ± SEM from Ca^2+^ transient amplitude (*n* = 12, *N* = 4, ^∗^*p* < 0.05, paired Student’s *t*-test), SCW latency (*n* = 9, *N* = 4, ^∗^*p* < 0.05, paired Student’s *t*-test), the percentage of cells exhibiting waves (^∗^*p* < 0.05, *N* = 4, Fisher’s exact test) and the caffeine-sensitive Ca^2+^ store load (*n* = 6 per group, ^∗^*p* < 0.05, Student’s *t*-test). **(D)** Representative recording of ROS production measured with MitoSOX in VMs. Signal was normalized to maximum fluorescence obtained on application of 10 mmol/L H_2_O_2_. VMs were treated with ISO and kaempferol and paced at 2 Hz (red bars) for 60 s. Graph in **(E)** depicts mean data ± SEM of normalized MitoSOX fluorescence (*n* = 7, *N* = 3, ^∗^*p* < 0.05, paired Student’s *t*-test).

We studied the effects of enhancing mitochondrial Ca^2+^ accumulation in TAB VMs on cytosolic Ca^2+^ cycling using Rhod-2, as illustrated in Figure 11. As with control VMs, the burst pacing-pause protocol was used to assess the propensity of arrhythmogenic Ca^2+^ release (Figure 11A). After treatment with kaempferol, TAB VMs has a significantly reduced Ca^2+^ transient amplitude (5.59 ± 0.71 Δ*F*/*F*_0_ ISO vs. 3.08 ± 0.42 Δ*F*/*F*_0_ ISO and kaempferol, ^∗^*p* = 0.002), as well as a approximately twofold in decrease in SCW latency (Figure 11C, ^∗^*p* = 0.001). The percentage of cells exhibiting SCWs was also significantly increased after kaempferol application (71% ISO vs. 100% ISO and kaempferol, ^∗^*p* = 0.01). These changes were accompanied by a significant decrease in SR Ca^2+^ content assessed by application of 10 mmol/L caffeine (Figure 11B, 5.46 ± 0.77 Δ*F*_caf_*F*/*F*_0_ ISO vs. 3.19 ± 0.30 Δ*F*_caf_*F*/*F*_0_ ISO and kaempferol, ^∗^*p* = 0.01). Decreased Ca^2+^ transient amplitude and reduced SR Ca^2+^ load is indicative of increased Ca^2+^ leak via hyperactive RyRs (Belevych et al., 2007; Terentyev et al., 2008).

**FIGURE 12:**
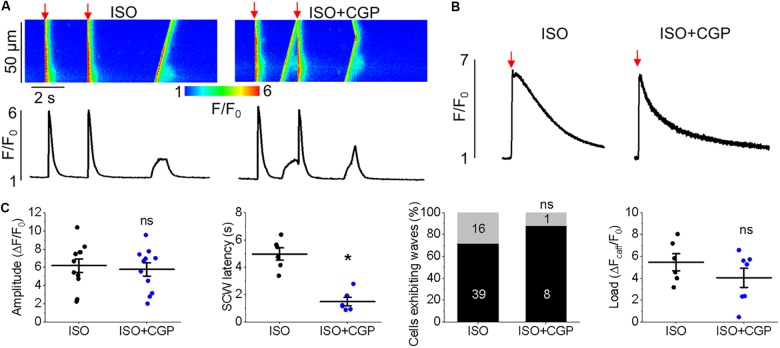
Enhanced mitochondrial Ca^2+^ accumulation by CGP-37157 promotes proarrhythmic SCWs in TAB rat VMs. **(A)** Representative confocal line scan images of Ca^2+^ transients and Rhod-2 fluorescence (*F*/*F*_0_) profiles of ISO treated (50 nmol/L) rat VMs undergoing 0.5 Hz pace-pause protocol before and after application of CGP-37157 (CGP, 1 μmol/L). **(B)** Representative traces of caffeine-induced Ca^2+^ transients (10 mmol/L). Graphs in **(C)** depict mean data ± SEM from Ca^2+^ transient amplitude (*n* = 11, *N* = 5, ns is not significant, paired Student’s *t*-test), SCW latency (*n* = 6, *N* = 5, ^∗^*p* < 0.05, paired Student’s *t*-test), the percentage of cells exhibiting waves (*n* = 18–55, *N* = 5, ns is not significant, Fisher’s exact test), and the caffeine-sensitive Ca^2+^ store load (*n* = 6, *N* = 5, ns is not significant, Student’s *t*-test).

**FIGURE 13:**
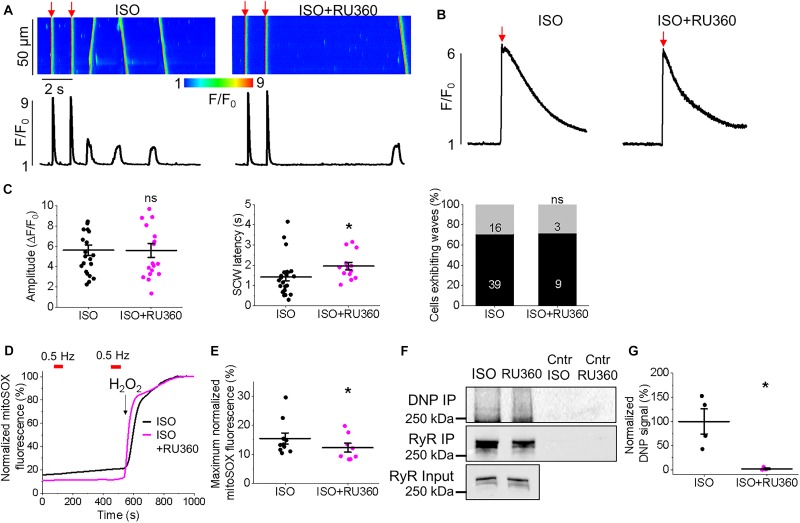
Block of mitochondrial Ca^2+^ uptake through MCU stabilizes RyRs in TAB rat VMs. **(A)** Representative confocal line scan images of Ca^2+^ transients and Rhod-2 fluorescence (*F*/*F*_0_) profiles of ISO-treated (50 nmol/L) TAB VM undergoing 0.5 Hz pace-pause protocol to induce spontaneous Ca^2+^ waves (SCWs), after 30 min. preincubation with Ru360 (2 μmol/L). **(B)** Representative traces of caffeine-induced Ca^2+^ transients (10 mmol/L). Graphs in **(C)** depict mean data ± SEM from Ca^2+^ transient amplitude (*n* = 19–22, *N* = 4, ns is not significant, Student’s *t*-test), SCW latency (*n* = 13–24, *N* = 4, ns is non-significant, ^∗^*p* < 0.05, Student’s *t*-test), the percentage of cells exhibiting waves (^∗^*p* < 0.05, Fisher’s exact test) and the caffeine-sensitive Ca^2+^ store load (calculated by application of 10 mmol/L caffeine, *n* = 6, *n* = 4, ^∗^*p* < 0.05, Student’s *t*-test). *N* = 4. **(D)** Representative recording of mitochondrial ROS production measured with MitoSOX in VMs. Signal was normalized to maximum fluorescence obtained on application of H_2_O_2_ (10 mmol/L). Myocytes were treated with ISO (black line) or ISO and Ru360 (pink line) and paced at 2 Hz (red bars). Graph in **(E)** depicts mean data ± SEM of normalized MitoSOX fluorescence (*n* = 7–10, *N* = 3, ^∗^*p* < 0.05, Student’s *t*-test). **(F)** RyR in freshly isolated control and TAB VMs was immunoprecipitated (IP) and immunoblotted for oxidation using DNP antibody. Representative images of DNP IP, RyR IP and RyR input signal from VMs treated with ISO or ISO and Ru360, with controls (Cntr). **(G)** Quantification of normalized DNP signal (%, *N* = 4 per group, ^∗^*p* < 0.05 vs. ISO, Student’s *t*-test).

Excessive ROS production is a hallmark of hypertrophy and we have previously established that in TAB VMs, there is increased mitochondrial ROS production in comparison to healthy controls which results in oxidation and thereby abnormally high activity of RyRs (Kim et al., 2017). In the present study, parallel experiments using the mitochondria-specific ROS indicator MitoSOX demonstrated that enhancement of mitochondrial Ca^2+^ accumulation with kaempferol further increased ROS emission in diseased VMs (Figures 11D,E). Signal was normalized to maximal fluorescence obtained upon application of 10 mmol/L H_2_O_2_.

Similar effects on cytosolic Ca^2+^ transients were obtained when enhancing mitochondrial Ca^2+^ accumulation in TAB VMs with block of NCLX via CGP-37157 (Figures 12A,B). There was a significant reduction in SCW latency (Figure 12C, 4.79 ± 0.38 s. ISO vs. 1.99 ± 0.45 s. ISO and CGP-37157, ^∗^*p* < 0.001), although no significant change in the SR Ca^2+^ content assessed by caffeine application was observed (Figures 12B,C).

### Inhibition of Mitochondrial Ca^2+^ Uptake With Ru360 Reduces ROS Emission and Increases Latency for Proarrhythmic SCWs

Given that application of MCU-inhibitor Ru360 attenuated triggered activity and arrhythmogenesis in TAB hearts *ex vivo* (Figure 1), we next assessed intracellular Ca^2+^ handling of VMs preincubated with 2 μmol/L Ru360 for 30 min. There were no significant differences in the Ca^2+^ transient amplitude and the percentage of cells exhibiting waves or the caffeine-sensitive SR Ca^2+^ content (Figures 13A–C). However, there was a significant increase in SCW latency, (Figures 13A,C, 1.42 ± 0.19 s. ISO vs. 1.96 ± 0.19 s. ISO and Ru360, ^∗^*p* < 0.001), indicative of stabilization of RyR-mediated Ca^2+^ release. Myocytes from TAB hearts displayed a decrease in MitoSOX fluorescence after preincubation with Ru360 (Figures 13D,E 15.46 ± 1.91% ISO vs. 14.93 ± 2.15% ISO and Ru360, ^∗^*p* < 0.001). There was also a significant reduction in oxidation of immunoprecipitated RyRs after treatment with Ru360 assessed using anti-DNP antibodies (Figures 13F,G). These data suggest that stabilization of SR Ca^2+^ release stems from attenuation of ROS emission by mitochondria and normalization of RyR redox state.

### Modifiers of Mitochondrial Ca^2+^ Uptake and Retention Do Not Alter the Velocity of SCWs

**FIGURE 14:**
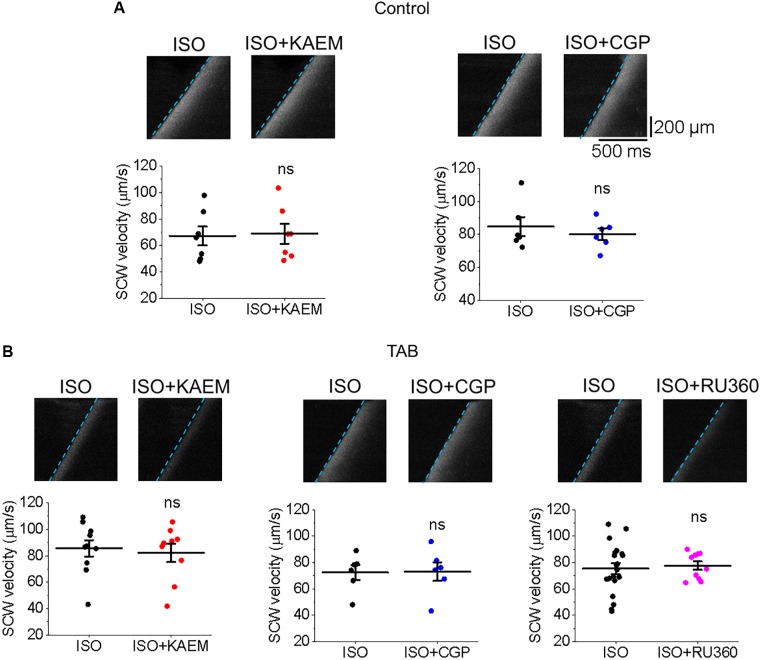
Enhancement or inhibition of mitochondrial Ca^2+^ accumulation does not modulate SCW propagation. **(A)** Velocity of SCWs in cultured control VMs. Graphs depict mean ± SEM (*n* = 6–9, *N* = 4–5, ns is non-significant, paired Student’s *t*-test). **(B)** Velocity of spontaneous Ca^2+^ waves (SCWs) in TAB VMs. Graphs depict mean ± SEM (*n* = 10–20, *N* = 4–5, ns is non-significant, Student’s *t*-test and paired Student’s *t*-test where appropriate).

Regenerative SCWs propagate via the ‘fire-diffuse-fire’ mechanism (Keizer and Smith, 1998; Maxwell and Blatter, 2012), whereby Ca^2+^ released from one cluster of RyR channels activates Ca^2+^ release from another. Increasing Ca^2+^ buffering can intercept Ca^2+^ diffusing from cluster to cluster and modulate SCW velocity (Ramay et al., 2010; Eisner et al., 2017), as was shown with SR Ca^2+^-ATPase (SERCa) enhancers (Fernandez-Tenorio and Niggli, 2018). It could be suggested that slower SCW wave propagation after enhancement of mitochondrial Ca^2+^ accumulation indicates a Ca^2+^ buffering capacity of mitochondria, serving as a sink for cytosolic Ca^2+^. However, neither the enhancement (with kaempferol or CGP-37157) nor attenuation (with Ru360) of mitochondrial Ca^2+^ accumulation altered the velocity of SCWs in either cultured control VMs (Figure 14A) or hypertrophic TAB VMs (Figure 14B). These data suggest that buffering capacity of mitochondria is insufficient to interrupt or slow SCWs, possibly due to concomitant changes in Δψm and local ROS.

## Discussion

The contribution of mitochondrial Ca^2+^ flux to myocyte excitation-contraction remains the subject of intense research, with both enhancement or reduction of [Ca^2+^]_m_ posited as therapeutic strategies to improve cardiac function and prevent arrhythmia in cardiac disease (Liu and O’Rourke, 2008; Kolhaas et al., 2010; Liu et al., 2014; Dietl and Maack, 2017; Schweitzer et al., 2017; Xie et al., 2018). Our present study provides evidence that enhanced mitochondrial Ca^2+^ accumulation dissipates Δψm and drives increased ROS in the mitochondria-SR microdomain. Subsequently, increased modification of RyR by ROS enhances channel activity and increases the propensity for proarrhythmic spontaneous SR Ca^2+^ release in the form of SCWs. This mechanism further exacerbates proarrhythmic triggered activity in hypertrophic hearts. Conversely, inhibition of MCU is protective against arrhythmogenesis, attenuating oxidative stress and reducing aberrant activity of RyR.

### Modulation of Mitochondrial Ca^2+^ and the Effects on Mitochondrial Function

The physical and functional coupling of the SR and mitochondria is critical for matching myocyte workload to mitochondrial ATP generation (Dorn and Maack, 2013; Lopez-Crisosto et al., 2017). The close association facilitates mitochondrial Ca^2+^ influx upon SR Ca^2+^ release (Sharma et al., 2000; Szalai et al., 2000; Csordás et al., 2001). To maintain Ca^2+^ flux balance in the steady state, uptake of mitochondrial Ca^2+^ is well matched to extrusion, so net [Ca^2+^]_m_ does not significantly change. We assessed mitochondrial Ca^2+^ uptake in cultured control and TAB VMs with the genetically encoded Ca^2+^ probe mtRCamp1h. In comparison to baseline, under β-adrenergic stimulation with ISO we observed an increase in [Ca^2+^]_m_ during periodic pacing (Figures 2B,E, 10A,C), likely to match the increased demand for ATP.

To modulate mitochondrial Ca^2+^ influx in cultured control VMs, we applied kaempferol or SB 202190 to directly enhance uptake through MCU, or CGP-37157 to inhibit NCLX thus attenuating Ca^2+^ efflux. Interestingly, application of these pharmacological agents did not increase [Ca^2+^]_m_, but instead altered the dynamics of Ca^2+^ accumulation or retention (Figures 2D,F). Similar effects were observed in TAB VMs treated with kaempferol and CGP-37157 (Figures 10B,D). The inability to significantly increase total [Ca^2+^]_m_ under these conditions points to the existence of limiting factors, preventing excessive mitochondrial Ca^2+^ loading in VMs.

There are three established mechanisms to prevent mitochondrial Ca^2+^ overload. Activation of mitochondrial Ca^2+^-activated K^+^ channels limits depolarization of the mitochondria, reducing the electrochemical inward driving force for Ca^2+^ (O’Rourke et al., 2005; Clements et al., 2015). Acidification of the mitochondrial matrix by an increased H^+^ flow through ATP-synthase has been shown to inhibit MCU (Moreau and Parekh, 2009). Additionally, increased matrix Ca^2+^ also promotes the opening of mPTP, dissipating Δψm and limiting [Ca^2+^]_m_ accumulation (Broekemeier et al., 1998; Hüser and Blatter, 1999; Elrod et al., 2010). This is evident in Figure 3, whereby application of kaempferol or CGP-37157 to cultured control VMs significantly depolarized Δψm. Interestingly, preincubation of VMs with mitoTEMPO, a specific mitochondria ROS scavenger (20 μmol/L, 30 min), did not prevent dissipation of Δψm by kaempferol (normalized TMRM fluorescence 89.7 ± 2.16% mitoTEMPO and ISO vs. 39.17 ± 3.59% mitoTEMPO, ISO and kaempferol, ^∗^*p* < 0.001, paired Student’s *t*-test, *n* = 19, *N* = 3), suggesting that this process is not ROS-dependent.

### Modulation of SR Ca^2+^ Release by [Ca^2+^]_m_ Is Mediated by Mitochondrial ROS

The RyR-mediated release of Ca^2+^ from the SR is critical to contractile activation. Termination of SR Ca^2+^ release allows for Ca^2+^ released into the cytosol to be resequestered and maintains the refractoriness of Ca^2+^ signaling during diastole (Terentyev et al., 2002; Szentesi et al., 2004; Sobie et al., 2005). Shortened Ca^2+^ signaling refractoriness due to hyperactive RyR increases the rate of SCWs in diseased myocytes (Belevych et al., 2012; Brunello et al., 2013), contributing to the pathogenesis of triggered arrhythmias (Pogwizd and Bers, 2004).

We assessed the effects of [Ca^2+^]_m_ modulation on Ca^2+^ handling and initiation of triggered activity at the whole organ level, using hearts from rats with TAB-induced hypertrophy, as illustrated in Figure 1. Application of MCU activator kaempferol reduced Ca^2+^ transient amplitude and exacerbated the proarrhythmic phenotype, with incidences of PVCs and spontaneous VT/VFs with higher VF frequencies. The focal activity and/or transmural outbreak-like activation patterns were frequently seen during PVCs and VFs, suggesting enhanced triggered activity may underlie exacerbated ventricular arrhythmias by kaempferol. In contrast the MCU blocker, Ru360, suppressed spontaneous VT/VFs. The activation maps of pacing induced VFs showed that rapid pacing caused conduction block and reentry formation, suggesting that the protective effect of Ru360 is most likely through suppressing triggered activity. In recordings of intracellular Ca^2+^ transients, it was evident that kaempferol and NCLX inhibitor CGP-37157 had detrimental effects on both cultured control VMs (Figure 4) and TAB VMs (Figures 11, 12), shortening SCW latency and increasing the propensity for spontaneous Ca^2+^ release, indicative of increased activity of RyR in both cultured control and hypertrophic TAB VMs. The experiments with RyR inhibitor dantrolene demonstrating attenuation of kaempferol-induced disturbances in Ca^2+^ cycling (Figure 6) further confirm the central role of dysregulated RyR-mediated Ca^2+^ release in this process.

Hyperactivity of RyR in cardiac disease is often attributed to posttranslational modifications, including phosphorylation of PKA- and CaMKII-specific sites, and oxidation of many reactive cysteines within the protein (Györke and Carnes, 2008; Niggli et al., 2013; Zima et al., 2014). Mitochondria are a major source of ROS in the myocyte and excessive ROS production is a hallmark of HF, hypertrophy and aging (Zima and Blatter, 2006), in parallel with perturbed Ca^2+^ homeostasis (Terentyev et al., 2008; Cooper et al., 2013; Kim et al., 2017). Mitochondrial-ROS signaling has also been suggested to directly modulate Ca^2+^ spark activity (Yan et al., 2008; Zhou et al., 2011). Our experiments using mitochondria-targeted ROS biosensor OMM-HyPer3 show that kaempferol induces surge in mito-ROS production (Figure 7). Furthermore, in the present study, Figure 8 clearly demonstrates increased local ROS in the vicinity of RyR and RyR oxidation in myocytes treated with kaempferol and CGP-37157. Importantly, while oxidative stress is already significant in hypertrophic myocytes, application of kaempferol further increased mitochondrial ROS emission in TAB VMs (Figure 11D), exacerbating Ca^2+^ mishandling.

Scavenging of mitochondrial ROS has been sufficient to alleviate the arrhythmogenic phenotype in multiple disease states (Mochizuki et al., 2007; Belevych et al., 2012; Cooper et al., 2013; Luo et al., 2013; Joseph et al., 2016) and normalize the redox state of RyR in TAB-induced hypertrophy (Kim et al., 2017). Importantly, inhibition of mitochondrial Ca^2+^ uptake with Ru360 was sufficient to attenuate Ca^2+^-dependent arrhythmia in *ex vivo* TAB hearts (Figure 1) and normalize Ca^2+^ homeostasis in isolated TAB VMs, stabilizing RyR-mediated Ca^2+^ release and attenuating proarrhythmic SCWs (Figures 13A–C). Furthermore, Ru360 reduced mitochondrial ROS emission assessed using mitochondria-specific ROS indicator mitoSOX (Figures 13D,E). This indicates that block of mitochondrial Ca^2+^ influx reduces mitochondrial ROS signaling in TAB VMs resulting in reduction of oxidation levels of RyR (Figures 13F,G). Our data suggest that increased mitochondrial Ca^2+^ accumulation facilitates increased mitochondrial ROS emission and the oxidation of RyR. This underlies enhanced RyR activity, increased spontaneous Ca^2+^ release in the form of arrhythmogenic SCWs, and a vicious cycle of Ca^2+^/ROS-induced myocyte dysfunction.

### Inhibition of NCLX-Mediated Mitochondrial Ca^2+^ Efflux Exacerbates Ca^2+^ Mishandling

During pathological mitochondrial Ca^2+^ overload, opening of mPTP offers an additional Ca^2+^ efflux pathway (Broekemeier et al., 1998; Hüser and Blatter, 1999; Elrod et al., 2010). Pharmacological inhibition of mPTP with cyclosporine A or genetic ablation of mPTP component cyclophilin D (that reduces opening) has shown to be protective against HF or ischemia-reperfusion injury (Griffiths and Halestrap, 1993; Hausenloy et al., 2010; Yarana et al., 2012; Gordan et al., 2016). However, there is ongoing controversy as to whether inhibition of NCLX can be protective in cardiac disease. In a guinea pig HF model, chronic inhibition of NCLX with CGP-37157 restored diminished [Ca^2+^]_m_, thereby improving redox homeostasis and protecting against arrhythmogenesis (Liu et al., 2014). In agreement with these findings, we recorded diminished mitochondrial Ca^2+^ transients in VMs from diseased hearts in comparison to controls (Figure 2 vs. Figure 10). However, incubation with CGP-37157 did not change significantly mitochondrial Ca^2+^ transient amplitude despite slowing down transient decay in TABs. It also did not improve but even worsened aberrant intracellular Ca^2+^ handling (Figure 12). Xie et al. (2018) reported an increased mitochondrial Ca^2+^ influx during diastolic period in mice with non-ischemic HF and posited that increased mitochondrial Ca^2+^ efflux drives activation of the sarcolemmal NCX and initiates EADs. In this work, inhibition of both influx and efflux were reported to have anti-arrhythmic effects. In direct contrast, the present study clearly demonstrates that inhibition of NCLX in both control and TAB VMs promotes proarrhythmic spontaneous SR Ca^2+^ release (Figures 4, 12). Importantly, knockdown of NCLX in a conditional loss-of-function mouse model caused severe myocardial dysfunction, HF and sudden cardiac death (Luongo et al., 2017). This was attributed to substantially increased ROS generation due to mitochondrial Ca^2+^ overload. Our results are in line with the latter and inhibition of NCLX with CGP-37157 significantly increased ROS emission in cultured control VMs (Figures 7, 8), resulting in defective SR Ca^2+^ handling in both cultured control (Figure 4) and TAB (Figure 12) VMs.

### Modification of Mitochondrial Ca^2+^ Influx and Efflux Does Not Affect SCW Propagation

While mitochondrial Ca^2+^ flux is closely associated with ATP generation and ROS emission, it has also been suggested that mitochondria may act as buffers that can shape global Ca^2+^ transients during EC coupling (Maack et al., 2006; Yan et al., 2008; Walsh et al., 2009; Drago et al., 2012; Zhao et al., 2013). Although the low affinity of MCU for Ca^2+^ (*K*d ∼ 10–20 μmol/L Ca^2+^; Bernardi, 1999) would limit uptake during diastole and normal Ca^2+^ transients, mitochondrial Ca^2+^ uptake could occur at high local [Ca^2+^] near SR Ca^2+^ release sites (Andrienko et al., 2009). One end of the mitochondria is in close proximity to these sites (∼37–270 nm; Sharma et al., 2000), and is physically tethered to the SR (García-Pérez et al., 2011; Chen et al., 2012) with strategic positioning of MCU near RyR (De La Fuente et al., 2016). This facilitates crosstalk between the organelles and tunneling of Ca^2+^ between the two has been reported in striated muscle (Shkryl and Shirokova, 2006). In cardiac disease, mitochondria-SR interfaces and therefore Ca^2+^ transport may be altered due to changes in expression levels of scaffolding proteins including Mfn1 and 2 (Dorn et al., 2015). We tested whether this occurs in our model of hypertrophy and did not find changes in expression levels of these proteins (Figure 9). Mitochondria may act as a buffer, serving as a sink of local Ca^2+^. Indeed, in a mouse model of CPVT, enhancement of mitochondrial Ca^2+^ influx reduced frequency of arrhythmogenic Ca^2+^ waves and incidences of VT/VF (Schweitzer et al., 2017). Also, Zhao et al. (2013) saw a reduction in the frequency of SCWs in myocytes treated with kaempferol after an FCCP-induced reduction in Δψm, while Ru360 increased SCW frequency.

Although SR Ca^2+^ release events are an important driver of [Ca^2+^]_m_, as the total mitochondria Ca^2+^ flux is small, the overall ability of mitochondria to shape intracellular Ca^2+^ dynamics remains debated (Dedkova and Blatter, 2008, 2013; O’Rourke and Blatter, 2009; Hohendanner et al., 2013; Williams et al., 2013; Eisner et al., 2017). Troponin I and SERCa are significant Ca^2+^ buffers in the myocyte, and enhancement or increased expression of SERCa has been shown to significantly improve Ca^2+^ buffering capacity and attenuate arrhythmogenic spontaneous Ca^2+^ release (Lyon et al., 2011; Briston et al., 2014). In recent work of Fernandez-Tenorio and Niggli (2018), specific enhancers of SERCa activity reduced the frequency and velocity of SCWs in mouse VMs. In the present study, enhancement of mitochondrial Ca^2+^ influx significantly increases the propensity for SCW in both cultured control and TAB VMs (Figures 4, 5, 11, 12), while neither enhancement or inhibition of [Ca^2+^]_m_ modulated the velocity and propagation of SCWs (Figure 14). Importantly, we do not find any differences in the rate of propagation of SCWs between control and TAB VMs where impairment of mitochondria to sequester Ca^2+^ leads to profound approximately fourfold decrease in the amplitude of pacing-induced mitochondrial Ca^2+^ transient (Figures 2, 10). These data are in line with previous studies that suggest while Ca^2+^ modulates mitochondrial function, mitochondria do not serve as a significant buffer of intracellular cytosolic Ca^2+^ (Bers et al., 1993; Negretti et al., 1993; Lu et al., 2013; Williams et al., 2013). The effects of increased ROS emission on intracellular Ca^2+^ homeostasis are likely overwhelming and cannot be compensated for by an increase in local Ca^2+^ buffering capacity by mitochondria.

## Conclusion

In conclusion, our data suggest that pharmacological enhancement of mitochondrial Ca^2+^ accumulation produces deleterious effects on Ca^2+^ homeostasis under β-adrenergic stimulation. It promotes excessive ROS that enhances RyR activity thereby proarrhythmic spontaneous Ca^2+^ release. In cardiac hypertrophy, where ROS defenses are weakened, it exacerbates the proarrhythmic alterations in Ca^2+^ handling. Inhibition of mitochondria Ca^2+^ uptake is protective because it reduces emission of ROS by mitochondria.

## Author Contributions

SH, B-RC, JO-U, GC, and DT participated in the study design. SH wrote first draft of manuscript. SH, RT, TYK, PB, RC, JO-U performed the experiments. SH, RT, TYK, B-RC, DT conducted data interpretation and analyses. SH, RT, TYK, PB, RC, JO-U, GC, B-RC and DT reviewed the manuscript submitted for publication. All authors revised and approved the final version of the manuscript.

## Conflict of Interest Statement

The authors declare that the research was conducted in the absence of any commercial or financial relationships that could be construed as a potential conflict of interest.
